# Extremity soft tissue coverage in the combat zone: use of pedicled flap transfers by the deployed orthopedic surgeon

**DOI:** 10.1186/s40779-020-00281-5

**Published:** 2020-10-24

**Authors:** Laurent MATHIEU, Soryapong PLANG, Nicolas de l’ESCALOPIER, James Charles MURISON, Christophe GAILLARD, Antoine BERTANI, Frédéric RONGIERAS

**Affiliations:** 1 Department of orthopedic, trauma and reconstructive surgery, Percy Military Hospital, 101 avenue Henri Barbusse, 92140 Clamart, France; 2 Department of surgery, French Military Health Service Academy, Ecole du Val-de-Grâce, Paris, France; 3 Department of orthopedic and trauma surgery, Edouard Herriot Hospital, Lyon, France

**Keywords:** Pedicled flaps, Limited resources, War surgery, Reconstruction, Training

## Abstract

**Background:**

In theaters of operation, military orthopedic surgeons have to deal with complex open extremity injuries and perform soft-tissue reconstruction on local patients who cannot be evacuated. Our objective was to evaluate the outcomes and discuss practical issues regarding the use of pedicled flap transfers performed in the combat zone on local national patients.

**Methods:**

A retrospective study was conducted on data from patients treated by a single orthopedic surgeon during four tours in Chad, Afghanistan and Mali between 2010 and 2017. All pedicled flap transfers performed on extremity soft-tissue defects were included, and two groups were analyzed: combat-related injuries (CRIs) and non-combat related injuries (NCRIs).

**Results:**

Forty-one patients with a mean age of 25.6 years were included. In total, 46 open injuries required flap coverage: 19 CRIs and 27 NCRIs. Twenty of these injuries were infected. The mean number of prior debridements was significantly higher in the CRIs group. Overall, 63 pedicled flap transfers were carried out: 15 muscle flaps, 35 local fasciocutaneous flaps and 13 distant fasciocutaneous flaps. The flap types used did not differ for CRIs or NCRIs. Complications included one flap failure, one partial flap necrosis and six deep infections. At the mean follow-up time of 71 days, limb salvage had been successful in 38 of the 41 cases. There were no significant differences between CRIs and NCRIs in terms of endpoint assessment.

**Conclusions:**

Satisfying results can be achieved by simple pedicled flaps performed by orthopedic surgeons deployed in forward surgical units. Most complications were related to failure of bone infection treatment. The teaching of such basic reconstructive procedures should be part of the training for any military orthopedic surgeon.

**Trial registration:**

Retrospectively registered on January 2019 (n°2019–090 1-001).

## Background

Modern conflicts present military surgeons with a high volume of extremity injuries requiring flap coverage [1]. Various studies have evaluated soft-tissue coverage outcomes in U.S. military patients following their evacuation from combat zones [1–4]. These reconstructive procedures were performed in specialized centers by multidisciplinary teams including orthopedic and plastic surgeons; such ideal care and management, however, is rarely accessible to local, national casualties, since they cannot be evacuated from the theater of combat [5]. Due to the lack of plastic surgeons in the battlefield, orthopedic surgeons often find themselves alone when managing local patients who present with complex extremity injuries requiring multi-tissue reconstruction [6–9].

In French forward surgical facilities, military surgeons treat both military personnel and local nationals not only as a result of combat-related trauma, but also in a humanitarian role providing medical support to the civilian population. Due to deficiencies in the local health system, these patients present in a variety of ways with, for example, recent trauma, older and neglected injuries, osteomyelitis or burns [10, 11]. In this austere environment, military orthopedic surgeons routinely perform soft-tissue coverage procedures using “simple, reliable and replicable” techniques [6–9, 12, 13]. However, the relevance of such reconstructive procedures can be questioned in infected cases when precise germ identification and extended appropriate antibiotic medication are not available [7, 8].

This study sought to evaluate and report upon pedicled flap transfers performed in combat zone medical treatment facilities (MTFs) using the evidence from one single surgeon during a variety of deployments. The hypothesis was that pedicled flap transfers are safe and useful procedures suitable for soft-tissue coverage within forward surgical facilities, even when the conditions of care are suboptimal.

## Methods

A retrospective study was conducted by a military orthopedic surgeon (LM) using data from their four deployments between 2010 and 2017 which consisted of two 3-month tours in Chad (2010 and 2011), one 3-month tour in Afghanistan (2012) and one 1-month tour in Mali (2017). All patients with an extremity injury requiring flap reconstruction were included. The study was approved by the appropriate institutional review boards.

Patients were managed by forward surgical teams (FST) in Chad and Mali, and in a combat support hospital (CSH) in Afghanistan. All facilities were equipped with operating theaters suitable for basic orthopedic surgery. The hospital capacity and microbiology laboratory facilities, however, were variable: the Afghan CSH and Chadian FST had 20 to 30 beds for impatient care, a well-equipped microbiology lab and an appropriate antibiotics endowment. The Malian FST, however, had only five beds for impatient care and limited laboratory services; antibiograms were not available and there were insufficient antibiotics available.

The following preoperative parameters were studied: patient demographics, the mechanism and location of the soft-tissue defect, and the eventual presence of primary infection. Operative parameters included the time from the trauma to soft-tissue reconstruction, number of prior debridement procedures, flap transfer type, and associated bone or tendon procedures. Three types of pedicled flaps were used: muscle flaps, local fasciocutaneous flaps, and distant (fascio) cutaneous flaps. Secondary division of the distant flap pedicle was carried out after 3 weeks. Time to additional skin grafting on muscle flaps or on the donor sites of fasciocutaneous flaps was also analyzed.

All postoperative complications were described according to the Clavien-Dindo classification [14]. Outcomes measured included flap loss, partial flap necrosis and early infectious complications. Flap loss was defined as a need for coverage-revision surgery. Partial flap necrosis was defined as necrosis that necessitated surgical debridement but did not require additional coverage surgery. Early infections were defined as a wound infection at the coverage site within 2 weeks of flap transfer that required a return to the operating theater. Endpoint assessment included the achievement of limb or finger salvage and late complications due to infection. Because of variable energy trauma and wound contamination, two groups of injuries were considered by the analysis: combat-related injuries (CRIs) and non-combat related injuries (NCRIs).

Data were collected using Excel (Microsoft Corp., Redmond, WA, USA) to calculate means ±standard deviations. A Student’s *t*-test was used for normal, continuous quantitative variables. Qualitative variables were compared using Fisher’s exact test. *P*-values of less than 0.05 were considered significant.

## Results

During the four-deployment study period, 41 patients (35 males and 6 females) with extremity soft-tissue defects were treated using pedicled flap transfers. The participants’ mean age at the time of surgery was 25.6 ± 15 years. The mechanism of injury was non-ballistic trauma in 18 cases, ballistic trauma in 15 cases, osteomyelitis in 6 cases and burning in 2 cases (Table 1). Only Chadian or Malian patients were treated for osteomyelitis and burn injuries. CRIs occurred significantly more frequently in Afghanistan (13/15 versus 2/15, *p* = 0.001).

**Table 1:** Injury patterns and distribution

	Open fracture	Osteomyelitis	Soft-tissue injury	Burn injury	Total
Knee & leg	18	4	1	–	23
Ankle & foot	1	–	2	–	3
Elbow & forearm	3	1	1	–	5
Hand	5	1	6	3	15
Total	27	6	10	3	46

Four patients presented with multiple blast lesions so in total 46 injuries required a flap reconstruction. Of these, 19 were CRIs and 27 were NCRIs. Most injuries were located on either the patients’ legs or hands, and there was a predominance of open fractures (Table 1). Twenty of the 46 injuries were infected. The average number of debridement procedures per injury was 1.8 ± 0.91 since serial debridement was required prior to flap coverage in 29 injuries. Negative wound pressure therapy (which lasted, on average, for 4.3 ± 2.5 days) was used between debridement sessions in 12 of these 29 injuries. Although primary infection tended to be more frequent in NCRIs (15/27 versus 5/19, *p* = 0.07), the mean number of debridement procedures required was significantly higher in the CRI cases (Table 2). Primary debridement and flap coverage were carried out simultaneously in 17 injuries.

**Table 2:** Treatment parameters and outcomes according to the injury mechanism

	CRIs	NCRIs	***P***-value
Prior debridement number, mean	1.5	0.8	0.05
Time to flap coverage^a^, mean (days)	25.5	34	0.45
Muscle flaps	5/15	10/15	1
Local fasciocutaneous flaps	14/35	21/35	0.43
Distant (fascio) cutaneous flaps	3/13	10/13	0.5
Flap loss	0/22	1/41	1
Partial flap necrosis	1/22	0/41	0.35
Early infection	3/19	3/27	0.68
Follow-up time, mean (days)	40	86.4	0.05
Limb salvage	17/19	26/27	0.56
Persistent bone infection	0/19	3/27	0.25

^a^ traumatic injuries only

**Fig. 1:**
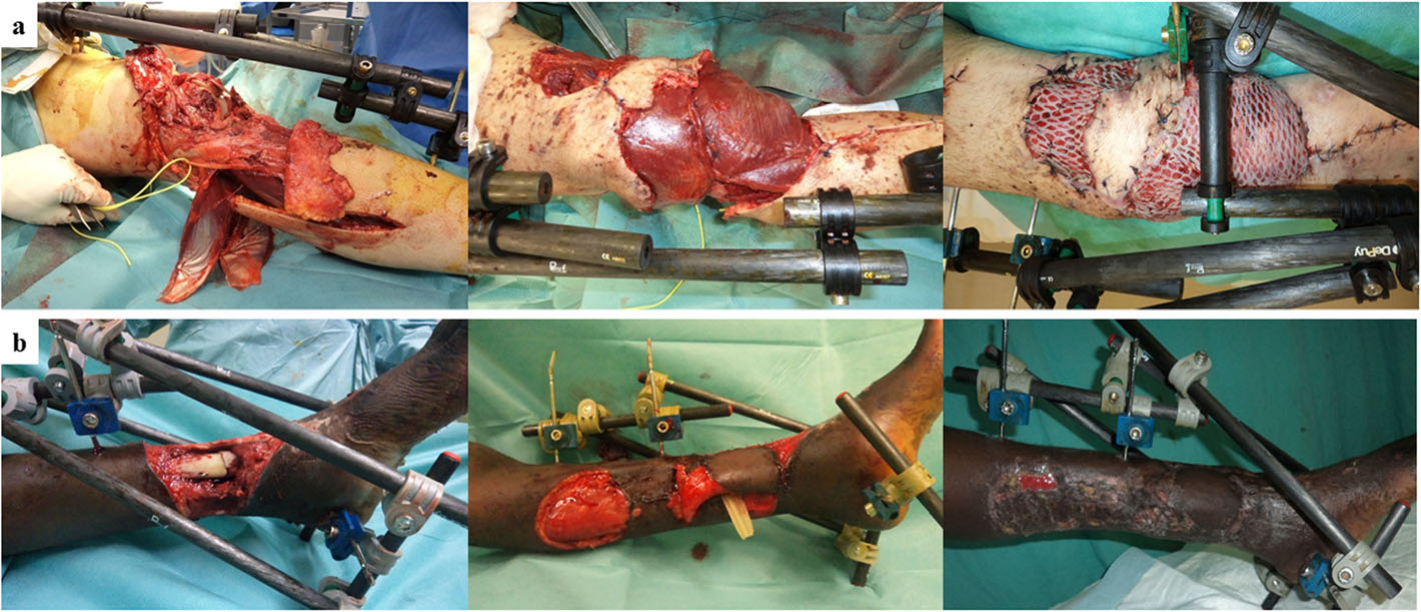
Locoregional flap transfers in the lower extremity. Simultaneous lateral and medial gastrocnemius flaps for a blast injury of the knee (**a**). Distally based great saphenous flap to cover a distal tibia open fracture in a 10-year-old patient (**b**)

**Fig. 2:**
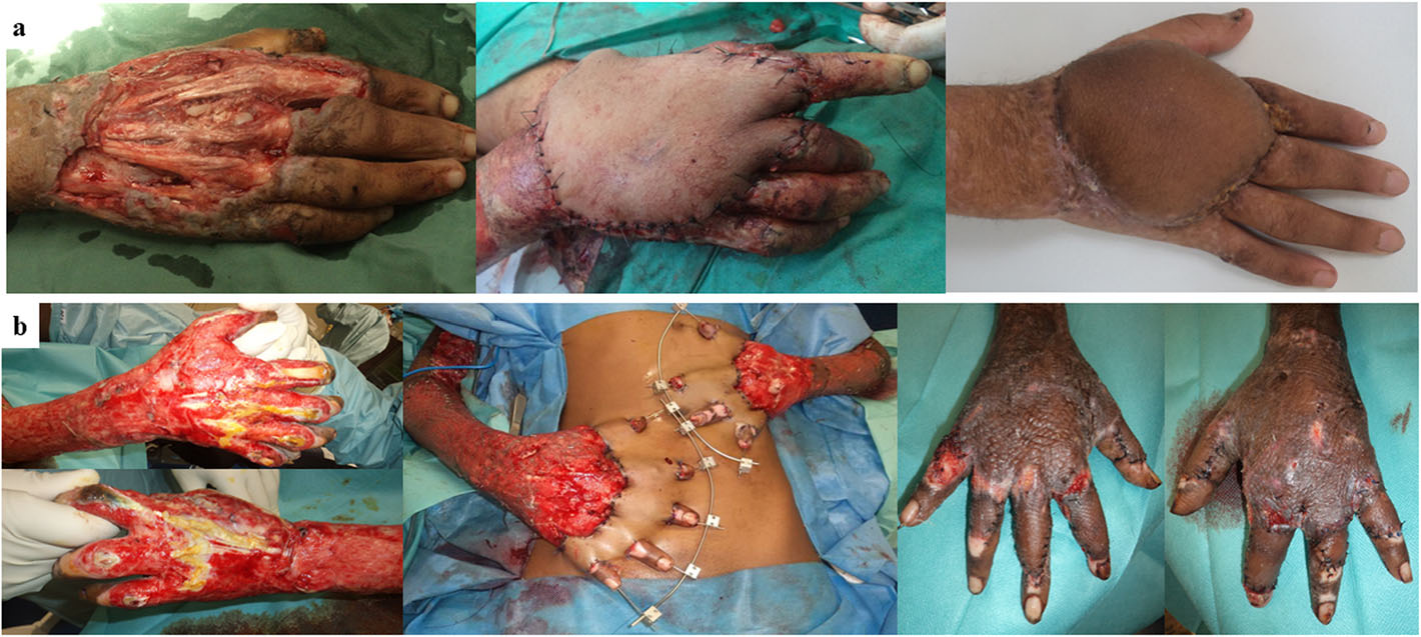
Distant fasciocutaneous flap transfers to the upper limb. Groin flap for a missile injury of the right hand: a flap repositioning was required after limited distal necrosis (**a**). Multiple abdominal flaps for a burn injury of both hands: note the flap loss on the right 5th finger (**b**)

Of the 36 traumatic injuries, there were no significant differences between CRIs and NCRIs in terms of the time from injury to flap surgery: the average was 29.5 ± 32 days (Table 2). Two locoregional flaps were combined in ten large post-traumatic defects, and nine simultaneous distant abdominal flaps were required to cover a burn injury of both hands in a single patient. Thus, a total of 63 pedicled flap transfers were performed: 15 muscle flaps, 35 local fasciocutaneous flaps and 13 distant fasciocutaneous flaps (Table 3). The flap types chosen for treatment did not differ for CRIs or NCRIs (Table 2). Muscle flaps were mostly used for proximal and mid-tibia coverage; distal tibia coverage was achieved by transposition and island fasciocutaneous flaps (Fig. 1). Three thumb reconstructions were carried out using island digital flaps. Distant flaps were exclusively used for hand and forearm coverage (Fig. 2). Additional skin grafting was required in 40 of the 63 flap transfers and performed after a mean delay of 5 ± 5 days. Associated procedures for bone and tendon reconstruction are detailed in Table 4.

**Table 3:** Flap distribution according to soft-tissue defect location

	Knee & leg	Ankle & foot	Elbow & forearm	Hand	Total
**Muscle flaps (*n*** **= 15)**					
Lateral gastrocnemius	2	–	–	–	2
Medial gastrocnemius	5	–	–	–	5
Soleus	6	–	–	–	6
Tibialis anterior	1	–	–	–	1
Latissimus dorsi	–	–	1	–	1
**Local fasciocutaneous flaps (*n*** **= 35)**					
Translation or rotational	11	2	1	9	23
Island flaps					12
*Distally based great saphenous*	*2*	*–*	*–*	*–*	*2*
*Distally based sural*	*3*	*–*	*–*	*–*	*3*
*Lateral supramalleolar*	*–*	*1*	*–*	*–*	*1*
*Medial plantar*	*–*	*1*	*–*	*–*	*1*
*Proximal radial*	*–*	*–*	*1*	*–*	*1*
*Digital island*	*–*	*–*	*–*	*4*	*4*
**Distant (fascio) cutaneous flaps (*n*** **= 13)**					
Abdominal	–	–	–	9	9
Groin	–	–	2	1	3
Thenar	–	–	–	1	1

**Table 4:** Procedures associated with flap coverage (debridement excluded)

Procedures	No
External fixation	21
Internal fixation	11
Cement spacer implantation^a^	10
Iliac bone grafting	4
Tendon repair	3

^a^ First step of the induced membrane technique

for bone defect reconstruction

There were nine type III Clavien-Dindo complications: one abdominal flap loss, one partial necrosis of a groin flap (Fig. 2a), one knee joint-fluid fistula (Fig. 1a) and six early infections. Other complications were type I: two minimal marginal losses of fasciocutaneous flaps and one dehiscence at the donor site of a groin flap. There were no differences between CRIs and NCRIs in terms of postoperative complications (Table 2).

The mean follow-up time was 71 ± 95 days for all cases and was superior for the patients with NCRIs because these were Chadian patients who were treated during the first tour (in 2010) and then reviewed during the second tour (in 2011). Otherwise, there were no significant differences between CRIs and NCRIs in terms of endpoint assessment (Table 2). Three patients in Mali were offered a late amputation due to severe persistent infection; one of them declined. Limb salvage was successful in 38 of the 41 cases. Three patients had a chronic pus-fistula under their muscle flap reconstruction related to tibia infection: two had been treated for a neglected open fracture and one for severe osteomyelitis.

## Discussion

Battlefield MTFs provide life- and limb-saving care through damage-control surgical procedures. The conditions in the field are rudimentary and, as such, may be ill-suited to support specialized plastic surgery. Therefore, studies reporting the results of flap reconstruction in theatres of operations are rare. Marchaland et al. [6] and Barbier et al. [8] analyzed the use of muscle and rotational fasciocutaneous flaps for recent, or neglected, open-tibia fractures, septic non-unions and osteomyelitis. We have also previously reported a series of 35 pedicled flap transfers performed for soft-tissue reconstruction of various CRIs in Afghanistan [7]. Klem et al. [5] reported on a case series of microsurgical free-flap procedures, performed by plastic or ear, nose and throat surgeons together with orthopedic surgeons, in U.S. CSHs that were deployed in Iraq and Afghanistan.

To our knowledge, this is the only study that has been published to date that reports only the cases of one single surgeon over four tours of duty in different locations. We believe that this focus on one practitioner in four difference MTFs enhanced the reliability of the results since it reduced the likelihood of confounding effects due to either inter-surgeon variability or conditions encountered in any one specific theater of combat or MTF.

In the ideal conditions of modern reconstructive units within well-provisioned healthcare facilities, specific soft-tissue reconstruction options are individually tailored to the wound, available and reliable flap sources, associated injuries and specific rehabilitation goals for each patient [1]. Within the limitations presented by the battlefield MTF environment, however, the choices for soft-tissue coverage methods are restricted. Factors that affect treatment decisions include the orthopedic surgeon’s expertise and available resources in terms of surgical equipment, antibiotics, laboratory analysis facilities or even the number of available beds [7, 8]. For these reasons, the simplest solution for coverage is always preferred [7]. In our experience, pedicled flaps combined with skin grafts allowed reconstruction of almost all presented soft-tissue extremity injuries, even large ones. Simultaneous local, or distant, pedicled flaps were used successfully in this cohort for massive injuries and defects as an alternative to free transfers [2, 7]. By contrast, the use of free flaps was never chosen. This decision was based not only on our limited experience with such procedures, but also on a conscious decision based on experience in the field which has indicated that free flaps are unsuitable for use as a treatment in combat zone MTFs for two reasons: the first is that free flaps require microsurgical techniques and specific post-operative care that can hardly be carried out by non-specialized healthcare teams. Secondly, free flap surgery requires an extended period in theater that can jeopardize the operational activity of a forward surgical facility [7]. Additional skin grafts were carried out together with flap transfers in most patients but were deferred in cases at risk of early infection recurrence.

In the present study, the reported pedicled flap transfer surgeries had a low failure rate for both CRIs and NCRIs. The overall success rate for flap coverage performed in the field was over 90%, a figure comparable to various other authors’ reports about war-related extremity reconstructions performed in patients after evacuation out of the combat zone [1, 3, 5, 15]. We have attributed this high success rate to the near-exclusive use of simple, reliable and replicable pedicled flap transfers, which were perfectly suited to both CRIs and NCRIs in a non-specialist surgical MTF [7, 12]. Transposition, or rotational, fasciocutaneous flaps and muscle flaps were the two types most often used, regardless of the injury location or cause. Since these flaps do not require pedicle dissection, they were easily performed by a surgeon who was not specialized in plastic surgery.

Other flap types were also represented in this study, however, such as perforator and distant flaps. Perforator flaps are not recommended for CRI treatment due to the extensive soft-tissue injury and the potential violation of fascial planes and perforators caused by the projectile’s kinetic energy [3]. They were mostly employed to treat NCRIs; they were dissected as island flaps with a large adipofascial pedicle (designed with a ratio L/l < 4) following doppler examination. When using such flaps, the non-specialized surgeon should be aware that the flap design is not only based on the location of the vascular territory of the perforator, but also on the perforator flow direction [16]. Propeller perforator flaps were never used because they were too technically demanding [17]. Distant flaps were indicated to salvage upper limbs, mostly at the hand level, even though they required a minimum 3-week hospital stay. By contrast, we did not use cross-leg flaps for tibia coverage as our experience has led us to conclude that an amputation should be considered in absence of available local flaps in such austere healthcare settings [12, 13].

Although we had only one complete flap failure, we experienced twelve flap complications, half of which were due to early infections. These results demonstrate the importance of infection control in both CRIs and NCRIs. Regardless of the method of soft-tissue reconstruction, adequate debridement of necrotic or infected tissue is critical for the overall success of any reconstructive modality [3, 15]. Highly contaminated war wounds usually require serial debridement and broad-spectrum intravenous antibiotics prior to definitive coverage [3]. Management of NCRIs, such as neglected open fractures, septic non-unions and chronic osteomyelitis, also follow the same therapeutic rules. Within the limitations of MTF conditions, therefore, treatment of bone infections should be undertaken with caution if extended courses of antibiotics, sequential procedures and close monitoring (for several months) will not be possible [10, 12, 13].

As mentioned above, and in agreement with the work of Kamath et al. [18], the findings from this study have clearly demonstrated how an orthopedic surgeon with the basic knowledge in local vascular anatomy was able to harvest an appropriate local, regional or distant pedicle flap and so successfully manage the majority of presented soft-tissue defects in the field. A supplemental training in reconstructive techniques would, however, be required for deployed orthopedic surgeons who are not familiar with extremity reconstruction. In the French Military Health Service such training is now included in the Advanced Course for Deployment Surgery [19]. During the training, basic pedicled flap transfers and bone reconstruction techniques that can be performed in healthcare facilities with limited resources are learned through lectures, hands-on exercises on cadavers and case studies [12].

This study has several limitations. The first is that the studied population was heterogenous in terms of factors like injury mechanism and the time elapsed before management. This was unavoidable when working in a precarious setting as a combat zone. Secondly, we acknowledge that indications for the different pedicle flaps may have been open for discussion as they reflected only the views and experience of one surgeon. Thirdly, the transient nature of the healthcare was also a regrettable and unavoidable consequence of the surgery being performed in a combat zone. Thus, the short follow-up made it impossible to evaluate long-term limb salvage, bone infection control and achievement of bone union.

## Conclusion

The original hypothesis of this study was that pedicled flap transfers are safe and useful procedures suitable for soft-tissue coverage within forward surgical units. Regardless of the treatment challenges from infection in such a setting, we believe that these data could support this hypothesis. It is, therefore, our considered opinion that all military orthopedic surgeons should be trained to perform such basic reconstruction techniques as, except perhaps in cases of pre-existing bone infection, these techniques permit limb salvage in the majority of open extremity soft-tissue injuries encountered in the field.

## Data Availability

The datasets analyzed during the current study are available from the corresponding author upon reasonable request.

## Abbreviations

CRI: Combat related injury
CSH: Combat support hospital
FST: Forward surgical team
MTF: Medical treatment facility
NCRI: Non-combat related injury
