# Correction to: Extremity soft tissue coverage in the combat zone: use of pedicled flap transfers by the deployed orthopedic surgeon

**DOI:** 10.1186/s40779-020-00290-4

**Published:** 2021-01-21

**Authors:** Laurent Mathieu, Soryapong Plang, Nicolas de l’Escalopier, James Charles Murison, Christophe Gaillard, Antoine Bertani, Frédéric Rongieras

**Affiliations:** 1 Department of orthopedic, trauma and reconstructive surgery, Percy Military Hospital, 101 avenue Henri Barbusse, 92140 Clamart, France; 2 Department of surgery, French Military Health Service Academy, Ecole du Val-de-Grâce, Paris, France; 3 Department of orthopedic and trauma surgery, Edouard Herriot Hospital, Lyon, France

**Correction to: Mil Med Res 7, 51 (2020)**

**https://doi.org/10.1186/s40779-020-00281-5**

Following publication of the original article [1], the editor identified the wrong version of the article’s published, the correct version is in the following page, the original paper has been updated.
